# Perceptual link between inadequate water, sanitation, and hygiene (WASH) stressors and common mental symptoms in Ethiopian health workers: A qualitative study

**DOI:** 10.1371/journal.pone.0314170

**Published:** 2025-01-10

**Authors:** Yitagesu Habtu, Abera Kumie, Medhine Selamu, Hidenori Harada, Morie Kaneko, Mirgissa Kaba, Eshetu Girma

**Affiliations:** 1 Department of Preventive Medicine, School of Public Health, Addis Ababa University, Addis Ababa, Ethiopia; 2 Department of Mental Health Epidemiology, School of Nursing and Midwifery, Addis Ababa University, Addis Ababa, Ethiopia; 3 Graduate School of Asian and African Area Studies, Kyoto University, Kyoto, Japan; 4 African Population and Health Research Center, Addis Ababa, Nairobi, Kenya; Universidad Santiago de Cali, COLOMBIA

## Abstract

**Background:**

Despite the rising prevalence of common mental symptoms, information is scarce on how health workers make sense of symptoms of mental disorders and perceive a link with inadequate water, sanitation, and hygiene (WASH) as work stressors to understand causation and produce useful knowledge for policy and professionals. Therefore, this study aimed to explore how health workers perceive the link between inadequate WASH and common mental symptoms (CMSs) at hospitals in central and southern Ethiopian regions.

**Methods:**

We used an interpretive and descriptive phenomenological design guided by theoretical frameworks. Three focus group discussions (FGDs) and 10 in-depth interviews with health workers were conducted. We explored the perceived link with inadequate WASH after assessing health workers’ conceptualization of common mental health symptoms. The interviews and FGDs were audio recorded, transcribed, and translated into English. Coding and categorizing were supported by MaxQDA software 2020. Thematic analysis was performed and themes were supported by participants’ quotes when necessary.

**Results:**

Four themes emerged from the data based on the guiding theoretical models relevant to the research questions. Most health workers believed and frequently suggested that inadequate water supply, functional hand hygiene, environmental cleaning, and waste management services at the points of care increased the risk of occupational stress, job anxiety, and occupational depression symptoms. Many participants believed that inadequate WASH would cause them to experience negative professional quality of life including burnout, compassion dissatisfaction, and compassion fatigue at work despite believing that WASH problems cannot be solved at the individual level. Participants recurrently listed many individual, organizational, and system-level barriers to reducing and coping with inadequate WASH as stressors.

**Conclusions:**

Healthcare workers perceived inadequate WASH components as contributing to symptoms of common mental disorders and negatively impacting their professional quality of life. They faced multiple barriers at individual, organizational, and health system levels, which hindered their ability to manage work-related stress and seek mental health support. The findings suggest the implementation of integrated WASH and mental health services for healthcare workers, ranging from establishing individual-level interventions to increasing access to WASH around care areas through collaborative efforts with healthcare administration, broader health systems, water supplies, and sewerage services.

## Introduction

Individual ways of making sense of the symptoms of mental disorders and their associations with specific stressors, including inadequate water, sanitation, and hygiene (WASH), are relevant to understanding causation with common mental symptoms and may provide useful knowledge for policy and professional practice. Despite recognising social determinants of mental health in general as a policy and research addenda [1], more evidence on the social determinants of mental illnesses for the working population, including health workers, is required [2]. Among the social determinants of mental health, inadequate water, hygiene, and sanitation (WASH), which are workplace stressors [1], are not well addressed. Persistent exposure to insecurity and inadequacy of WASH as mental health facilities, and infrastructures at the point of care continue to cause healthcare functional disruptions in low-income countries [3] due to its need for high investment. This may cause increased job demand and decreased job resources, including health workers’ capacity to control their jobs, and copping up of the problem, and finally put health workers under psychological distress, depression and general anxiety based on the previous theories of stress such as a transactional model of stress (TMS) [4] and the job demand resources framework(JDR) [5].

Achieving adequate water, hygiene, and sanitation services at the point of healthcare is a global agenda as a key infrastructure and resource input for infection prevention and control [6]. Despite focusing on somatic health impacts, such infrastructures are equally important for the mental health and well-being of the working population to the occupational health hazards points of view. Globally, in 2021, half of the healthcare facilities lacked basic hygiene services (such as a lack of water, soap, or alcohol-based hand rubs in patient care areas and 5 metres around toilets) [6]. This impacts about 688 million individuals who receive care at facilities [6]. Over one-third had no access to water on site, affecting 857 million people who received care at facilities. Basic sanitation and environmental cleaning were available to only 7% of the global population, while waste management services were limited to 24%. Additionally, 78% of healthcare facilities faced high service interruptions in their improved water source facilities [6]. In connection with a lack of water, poor sanitation, and poor hygiene at delivery points, health workers could face low-performance achievements, low job satisfaction, increased fear of infection in care areas, fear causing patients to hospital-acquired infections due to working in unhygienic conditions, and feelings of discontented work environment, which could ultimately increase the risk of contracting mental symptoms. Low-income countries including Ethiopia faced ow coverage of basic water (62%), satiation (36%), hygiene (21%), waste management (73%) and environmental cleaning (5%) for healthcare facilities [6] in 2021. A study of healthcare facilities in Ethiopia’s capital found that 100% had limited sanitation access, 88.4% had limited and 3.5% had no access to hand hygiene services, 97.7% lacked environmental cleaning services, and 29% lacked waste management services [7].

Inadequate WASH, as a stressor, may play an important role in the psychosocial causal pathways of common mental symptoms, as supported by the earliest original biopsychosocial health model [8] and recent explanatory suggestions [9] for mental disorders. Therefore, studies exploring the link between WASH-related stressors and mental distress potentially add valuable evidence to the current scientific understanding. Such perceived causal relationships between inadequate WASH and common mental symptoms could be explored using qualitative approaches like other qualitative causal explorations [10, 11] using interpretive-constructive instances. We one theoretical framework to explore health workers’ conceptualization of common mental health symptoms, self-identification as having common mental health symptoms (SICMS) [12] before perceptual causal explanation, and two other theoretical models of stress to perceptual causal explanations, a transactional model of stress (TMS) [4] and the job demand resources framework (JDR) [5].

The SICMS originated from the Health Belief Model (HBM) [13] to understand an individual’s awareness of symptoms in the context of a disease including mental illnesses. The SICMS) constructs guided our study on how to explore health workers’ awareness about symptoms, perceived vulnerability, perceived experiences, perceived causal like of work stressors with CMSs, and perceived controllability and or preventability of occupational stress, occupational anxiety, and occupational depression. Within the SICMS framework, perceived meaning or awareness of symptoms (stress, depression, and anxiety) was judged against DSM-5 [14] for anxiety and depression [14, 15]. Then, the perceived causal link with the specific work-related stressor (s) raised by the health workers was also explored as described under the operational definition section. For occupational stress, a reflection of various somatic and mental symptoms for an extended duration (6 months or more) followed by the ability to provide the link between work stressors and stress with the duration and frequency of occurrence was explored [16].

The TMS was developed by Lazarus and Folkman [4] explaining coping with stressors involving appraisal, coping strategies, moderators, and outcomes. According to TMS, primary appraisal assesses stress relevance, while secondary appraisal analyses the ability to manage stress. Coping strategies encompass cognitive and behavioural efforts, moderators include information seeking and social support, and outcomes reflect coping effectiveness. When coping with stressors fails and is perceived as threatening, individuals may exhibit somatic, physical, and behavioural symptoms in response to stressors. Regarding TMS, the exposure to workplace stressors (in this case, inadequate WASH), individual psychological factors (in this case, the appraisal and coping skills of health workers), and psychosocial factors (in this case, failure to access WASH-related resources at the point of healthcare) could increase mental reactions. If health workers feel unable to cope with the inadequacy of WASH, they may perceive the challenge as beyond their control, and continue to fear consequences such as being infected and fear of infection risks to the patients they care for risk. Consequently, they experience somatic and mental symptoms leading to chronic distress.

The latter theoretical model was proposed by Bakker [5] and states that if a worker is exposed to greater job demands (both psychological and physical demands) and has less control over the demands due to resource constraints (physical, psychological, and emotional resources) to cope with a stressful environment, the individual starts to experience distress, somatic symptoms. According to this theory, inadequate WASH components in the care areas could be considered kinds of job stressors taxing health worker’s physical, psychological, and social demands, and failure to accomplish tasks related to patient care, and reduced job control capacity causing chronic psychological distress. Where there are theoretical explanations, studies explicitly explaining this plausibility are limited to both global and low-income settings including Ethiopia.

How health workers appraise inadequate WASH-related stressors matters most, and depends on health workers’ perceptions to predict mental reactions or symptoms according to the TMS [4]. Accordingly, health workers may appraise exposure to inadequate WASH in care areas as a risk for infection, injury, care dissatisfaction, poor performance, and failure to achieve positive medical outcomes for the patient. As a result, the greater demand and inability to control these conditions due to resource constraints [5] may increase the risk of developing common mental health symptoms (CMSs), including chronic stress, anxiety, and depression. In other words, if health workers once appraise inadequate WASH components as threatening events, they may adopt individual coping strategies to achieve relief [17]. The strategies may include rational task-oriented behaviours, emotional release, use of home/individual resources to substitute, postponing action by distracting attention, and passive attempts to tolerate the effect, as suggested by Dewe [18] despite the need to supplement the new context with more options. Hence, studying the causal perceptions of health workers about ‘inadequate WASH’ as a stressor to their health and performance-related issues is a necessary step toward promoting health behaviours, coping strategies, educational interventions, and implementing other individual- and organizational-level interventions.

Globally, studies have revealed that a significant proportion of health workers experience persistent distress (37%), anxiety (40%), and depression (37%) [19]. In sub-Saharan Africa, a meta-analysis indicated that 35.7% to 82.5% of health workers are affected by depressive symptoms. In Ethiopia, studies during the coronavirus disease-19 (COVID-19) era reported a mean prevalence ranging from 12.4% to 61.9% for high-level perceived stress, 21.9% to 78% for anxiety, and 20.2% to 60.3% for depressive symptoms [20–23]. A recent study [24] also indicates a higher prevalence of work-related depressive symptoms, job anxiety, and occupational stress symptoms. Keeping other confounders in mind, higher depressive symptom prevalence is observed in facilities with lower national WASH coverage according to comparisons made by taking the national health facilities’ coverage of WASH [6] and taking the mean prevalence of depressive symptoms from a meta-analysis study [19]. Despite no studies on the healthcare population, a study from other populations also revealed a significant association between water insecurity and inadequate sanitation and common mental disorders, including psychological distress, anxiety, and depression [25].

In Ethiopia, where there is a serious inadequacy of WASH-related infrastructures at the points of care of the majority of healthcare facilities, there is a scarcity of information on how health workers perceive exposure to inadequate WASH as stressors increase the risk of common mental symptoms, perceive the impact of inadequate WASH on the professional quality of life, cope with inadequate WASH, and identify barriers to reducing inadequate WASH as stressors. Because health workers are expected to have better mental health literacy, exploring how WASH conditions are interpreted, felt, appraised, and linked to mental health symptoms provides insights to policymakers and implementers for better integration of inadequate WASH and other mental health services at work. Although intervening in such infrastructures requires the involvement of several sectors, the way health workers subjectively evaluate how they can reduce and cope with the situation matters whether health workers develop persistent psychological and physical responses and further develop workplace anxiety and depressive symptoms. Therefore, this study aimed to explore the perceived impact of inadequate water, sanitation, and hygiene (WASH) on mental health symptoms in health workers.

## Methods

### Study settings and period

We conducted this study among health workers at eight public health facilities in the Central Ethiopia Region and One Southern Ethiopia Region from 15^th^ January to 28th February 2023. The first in-depth interviewees were recruited on Sunday 15th January 2023, and the first FGD discussants were recruited on 17 January 2023. The last in-depth interviewee was recruited on 27 Monday, and the last FGD discussant was recruited on Tuesday 28 February 2023.

This qualitative study was conducted in randomly selected healthcare facilities (primary hospitals, general hospitals, and tertiary hospitals) for the quantitative study of a larger PhD project having both quantitative and qualitative objectives to address research questions, from different perspectives, and ultimately integrate the findings. Therefore, this study is one of the five PhD project studies on the same target population of the study setting. Among the other work-related stressors explored, WASH-related stressors and their link with common mental symptoms among healthcare workers are addressed within this study. The healthcare facilities selected for the study, the total estimated population, the population characteristics, and other study settings are described elsewhere [24].

Despite no information for the study area regarding related stressors, it is believed that the challenge would not differ from what we could observe in the context of low-income countries [6]. For example, the studies from the capital city of the country found that 100% had limited sanitation access, 88.4% had limited and 3.5% had no access to hand hygiene services, 97.7% lacked environmental cleaning services, and 29% lacked waste management services [7].

### Study design

We used interpretative phenomenological and descriptive qualitative design guided by theoretical frameworks. As explained in the introduction section of this work, the theoretical frameworks to guide explanations for the link between WASH components as stressors and common mental health symptoms, and professional quality of life were the TMS [4] and the JDR models [5]. To discuss the work-ascribed forms of common mental symptoms, we first explored how participants conceptualize common mental symptoms (stress, anxiety, or depressive symptoms using another theoretical framework, self-identification as having common mental health symptoms (SICMS) [12] before trying to explore their link with WASH related stressors. This was believed to help the participants interpret the symptoms, and how those symptoms may be linked to the current stressors of interest (WASH as work stressors). Although they are health professionals, this step was considered because participants may not have had adequate information about how those symptoms of mental health disorders were conceptualized and our discussion smoothly addressed the main objective of the study. Secondly, participants’ or colleagues’ experiences of stressors including inadequate WASH were also discussed during our interview and discussions. Thirdly, participants’ understanding of how each component of WASH (as stressors or risks or perceived causes) and common mental symptoms (outcomes) are linked was explored with their interpretation or meanings assigned to the stressor and perceived symptoms.

### Study participants and sampling strategy

Participants were selected from various units of the selected hospitals using a purposive sampling procedure. The initial stage involved the identification of departments or pinpoints in the health facilities (HFs) selected for PhD study with peer guides from the hospital’s matrons and medical directors.

We purposely selected healthcare workers who were working in three strata(primary hospitals, general hospitals, and specialized or teaching hospitals) of healthcare facilities—from different professional categories. Participants of in-depth interviews were purposely selected if they had 2 or more years of work experience, engaged in clinical or paramedic activities, held managerial positions such as directors or unit heads or managers or coordinators, or held ward heads or pinpoints from health workers at different health facilities. These in-depth interview participants were selected assuming that they have more understanding and experience with workplace mental well-being approaches and policy and readiness to provide occupational mental well-being services and resilience strategies at work. Ten healthcare workers for the in-depth interview to obtain rich information about the inadequacy of WASH-related stressors and their perceived link with common mental health symptoms.

Similarly, focus group discussants were purposely selected based on the representativeness of healthcare cadres such as physicians, nurses, midwives, laboratory technologists, pharmacists, and other paramedical health workers, to collect information on how health workers conceptualize occupational stress, occupational depression, occupational anxiety, and subjective exposure to WASH related stressors, and other work-related stressors in each cadre. We conducted 3 focus group discussions with eight study participants involved in each focus group discussion assuming that all health workers share a common understanding of stressors and related mental feelings despite having different speciality groups and different magnitudes and severities of stressors until we found repeated information.

### Data collection procedures

Interviewer guides for in-depth interviews (IDIs) and focus group discussions (FGDs) were developed and translated into the Amharic language. We trained two research assistants who had experience in assisting qualitative research with expertise in the health profession: one with an MSc degree in community psychiatry and another with an MPH in epidemiology to moderate focus group discussions. Two research assistants for taking notes and recording audio of the focus group discussion were recruited. Research assistants and one of the investigators of the study conducted in-depth interviews.

Moderators, under the guidance of the matrons and medical directors of the selected hospitals, recruited FGD and IDI participants based on the selection criteria. Moderators explained the purpose of the study, the selection process of respondents, the norms followed during the discussion, assured confidentiality, agreed, assigned participants, obtained written informed consent, and checked the audio recorder. Field notes, summary notes, and expanded scribbles of IDIs and FGDs were prepared and submitted daily by moderators and audio recorders, respectively. The recorded audio and background of the participants were submitted to the researcher at the end of each interview.

During the in-depth interviews and focus group discussions, all participants were invited to reflect on the symptoms of stress, depression, and anxiety. After exploring the perceived and actual definitions and symptoms of these common mental health symptoms, participants were also invited to explain how each symptom was associated with “occupational factors or stressors” to label them as “occupational stress”, “occupational depression”, and “job anxiety”. Once participants discussed those symptoms, they were invited to list possible work-related stressors including WASH-related stressors, and requested to perceptually link with at least one symptom of each common mental health symptom. Upon immediately mentioning specific work-related stressor(s) including the extent of inadequate WASH at the points of care, we proceeded to explore the perceptions of healthcare workers on how WASH-related components as a stressor(s) possibly cause at least one symptom of specific common mental health symptoms to themselves or their colleagues. Similarly, study participants were also invited to link WASH-related components as a stressors with components of professional quality of life (PQoL) by explaining to them the components of PQoL as burnout, compassion fatigue, and compassion satisfaction throughout the discussions and interviews.

### Operational definitions

**‘Inadequate WASH’** for this study applies if participants listed limitations or inaccessibility or unavailability of at least one basic WASH services at the points of care areas according to the WHO’s and UNICEF’s standards for [6]. These include water for cleaning and keeping hygiene; functional hand hygiene facilities (water, soap, alcohol-based hand rub, sanitizers, etc.) within 5 meters of toilets at points of care areas; environmental cleaning services (availability of cleaning protocols with trained staff and required supplies); and waste management services (waste segregation into at least 3 bins, safe disposal of sharps and infectious wastes) at the points of care areas (a place where health workers, patients, diagnosis and treatment, and visitors are located). The study participants’ accounts of their subjective experiences with WASH and related stressors at work were also considered in our study despite having difficulties in a clear understanding of the basic WASH standards at points of care.

**Common mental symptoms** (CMSs) are health workers’ subjective feelings or reflections of colleagues’ experience of mental and somatic symptoms related to the 3 common mental health issues hereafter termed ‘occupational stress’, ‘occupational depression’, and ‘occupational anxiety or job anxiety’ at work:

1. **Occupational stress** was defined as the ability of health workers to mention various somatic and mental symptoms for chronic or an extended duration (6 months or more participants’ perceived chronicity of the symptoms if difficulty in the duration of recall faced during our discussion or interview), and perceived inability to control work-related stressors (feeling of high workload or high job demand and low control (low resources)) according to the perceived occupational stress symptoms [16]. Accordingly, those health workers reflecting the perceived symptoms of all the new perceived occupational stress symptoms and able to provide the link between work stressors [16] with the duration and frequency of occurrence were coded as having “better comprehension of the meaning of occupational stress”. Whereas health workers who mentioned “high job demand versus low resources or low control or low social support (in their own wordings understanding)” irrespective of other symptoms were coded as having “low understanding of symptoms” of occupational stress.
2. **Occupational depression** or **work-ascribed depression** refers to health workers’ ability to mention cardinal symptoms of depression based on the Diagnostic and Statistical Manual of Mental Disorders (DSM-5) **(S1 File)** for 2 or more weeks. Participants’ perceptions of the symptoms were also considered if they couldn’t recall specific symptoms with duration. The ability to perceptually link at least one symptom of depression with any WASH-related stressors they mentioned was considered to assume occupationally linked depressive symptoms as defined in the new occupational depression definitions [15].
  Accordingly, those health workers who were able to mention five or more symptoms out of nine cardinal symptoms of depression and were able to link them with any work-related stressors were coded as having “awareness about symptoms of occupational depression”. Those health workers who had mentioned fewer than five symptoms but were able to link them with any work-related stressors were coded as having “low awareness about symptoms of occupational depression”. Those health workers who were able to list five or more cardinal symptoms of depression but who perceived that symptoms could not be completely linked to work-related stressors were coded as “did not believe depression is not linked with work-related stressors”. Health workers who were not able to mention at least one cardinal symptom were coded as “has no awareness about occupational depression”.
3. **Job anxiety or occupational anxiety for this study refers to** health workers’ ability to mention cardinal symptoms of generalized anxiety based on DSM-5 (S1 File) as a result of exposure to any work-related stressors including WASH-related stressors. Then, health workers were classified as “aware of occupational anxiety or job anxiety” if they could perceive the link between each symptom with work-related stressors they perceived or hypothetical views. The summarization is the same as that for occupational depression.

The remaining important perceptual opinions of health workers, such as vulnerability to, subjective experiences of, and perceived causal links between work stressors, including inadequate WASH, and perceived controllability of common mental symptoms, were assessed based on theoretical constructs used to understand health workers’ self-identification as having common mental health symptoms [12].

**The perceived link between inadequate WASH as stressors and CMSs** for this study was explored after participants’ subjective definition of CMSs and how they reflected the link based on either how they appraise inadequate WASH as threatening and inability to cope with and continue to be afraid of infection to themselves and their patients, fear of the poor medical outcome of patients they care for and fear of reduced performance [4] based on TMS theoretical framework [4] and/or [2] exposure to inadequate WASH and perceived inability to control the demand due to resource constraints based on the JDR framework [5].

### Data processing and analysis

The primary investigator transcribed, coded, and categorized all the audio recordings in the Amharic language and translated them into English. Moderators and/or note-takers and one of the investigators submitted the expanded scribbles, observations about WASH and related stressors at the points of care areas, and summary notes. Observations, scribbles, and field memos were cross-validated and merged into the corresponding transcripts. For significant information that seemed to be confusing, phone clarification was sought from the participants through contacts previously documented with the researcher during fieldwork. Then, translated transcripts were imported to MaxQDA 2020 software for coding and categorizing codes or creating themes.

Codes were developed as a coding frame from lists of constructs of the theoretical frameworks used for significant information. Both descriptive and interpretive coding were used for a single segment when necessary. Once significant information was obtained from the transcripts, themes emerged from categorizing codes containing the significant data. Two community mental health experts (MSc in community psychiatry) were briefed about the theoretical frameworks used in the study and invited to check a sample of codes to identify themes to validate the primary investigator’s categorization. After a discussion with the coder, corrections were made to some of the coding processes. We invited one member from each focus group discussion and four in-depth interviewees to check whether summaries reflected what had been discussed with participants and the realities among health workers to increase the trustworthiness of the information. Finally, we narrated findings under each theme and supported them with participants’ quotes when necessary. The findings are reported according to standards for reporting qualitative research (SRQR) [26] (S2 File).

### Ethical considerations

This study was approved by the Addis Ababa University College of Health Sciences-Institutional Review Board (CHS-IRB) as a PhD study under protocol number 080/22/SPH. Participants were made aware that their participation in the conversation and interview was entirely voluntary, and they were given the freedom to leave at any moment and to choose not to reflect any ideas they felt uncomfortable with. To maintain confidentiality, the FGD participants were given pseudonyms as “P1, P2, P3…”, and in-depth interviewees were given “IDI1, IDI2, IDI3…”. We used such pseudo-naming for each in-depth interviewee and discussant on the transcript and during the MaxQDA2020 software’s coding and analysis process. The data file (audio and transcripts) was also saved on a password-protected personal computer, and only the researchers had access to them. Based on the recommendation from the Ethical Review Board, reimbursement costs for expenses such as communication and transport were covered for each participant at the end of the discussion or the interviews.

## Results

### Sociodemographic characteristics of the study participants

Ten in-depth interviews and three focus groups involving thirty-four health workers were conducted. Ten and twenty-four health workers from various professional categories participated in the in-depth interviews and focus group discussions, respectively. The age of focus group discussants ranged from 28 to 55 years, with an average age of 32 years. Similarly, the age range of participants in the in-depth interview was between 30 and 41 with an average age of 35 years. Many of them were males (24 participants). The majority of participants were married (26 participants) had a Bachelor of Sciences (BSc) degree (26 participants), and had less than 10 years of working experience (23 participants), and were followers of protestant religion (20 participants) (Table 1).

**Table 1:** Sociodemographic characteristics of study participants in Central Ethiopia Region, February 2023.

Sr.No	Participant ID	Sex	Age	Religion	Marital Status	Education level	Work experience in years	Occupation
1	FGDP1	Male	55	Protestant	Married	BSc Nurse	34	Nurse
2	FGDP2	Male	36	Protestant	Married	Bsc Pharmacy	10	Clinical pharmacy
3	FGDP3	Female	29	Protestant	Married	BSc Midwife	10	Midwifery
4	FGDP4	Female	33	Orthodox	Married	BSc Midwife	10	Midwifery
5	FGDP5	Male	32	Orthodox	Married	BSc Nursing	11	Nurse
6	FGDP6	Male	33	Protestant	Married	BSc Nurse	4	Nurse
7	FGDP7	Male	28	Protestant	Married	BSc Medical Laboratory	8	Medical laboratory
8	IDI1	Female	37	Protestant	Married	MPH & Clinical Nurse	14	Nurse and pinpoint
9	IDI2	Female	36	Protestant	Married	BSc Nurse	16	BSc Adult Nursing
10	IDI3	Male	36	Protestant	Married	BSc Nurse	14	BSc Adult Nursing
11	FGDP8	Male	28	Orthodox	Married	BSc Midwife	10	BSc in Midwifery
12	FGDP9	Male	29	Muslim	Married	BSc Pharmacy	2	BSc in Pharmacy
13	FGDP10	Male	28	Orthodox	Married	BSc Nurse	7	BSc Nurse
14	FGDP11	Male	29	Muslim	Married	BSc Laboratory	9	BSc laboratory
15	FGDP12	Male	31	Muslim	Married	Radiology Nurse	7	Diploma Nurse
16	FGDP13	Male	31	Muslim	Married	BSc PH	7	BSc Public Health
17	FGDP14	Male	33	Muslim	Married	MD	4	General Practitioner
18	IDI4	Male	34	Orthodox	Single	BSc Psychiatry	5	BSc Psychiatry community
19	IDI5	Male	35	Muslim	Married	Nursing	8	BSc Nurse
20	FGDP15	Male	31	Orthodox	Married	Pharmacy	15
21	FGDP16	Female	28	Protestant	Married	Clinical Nurse	7	BSc Nurse
22	FGDP17	Male	28	Protestant	Single	BSc Laboratory	3	BSc laboratory
23	FGDP18	Male	29	Protestant	Single	BSc Midwife	2	BSc Midwifery
24	FGDP19	Male	30	Protestant	Single	MD	3	MD
25	FGDP20	Male	31	Protestant	Single	BSc Psychiatry	2	BSc Psychiatry community BSc Psychiatry
26	FGDP21	Male	32	Protestant	Married	BSc Neonatology Nurse	9	BSc Neonatology
27	FGDP22	Male	36	Muslim	Married	BSc Nurse & BSc PH	13	BSc Nurse/& BSc HO
28	FGDP23	Female	42	Protestant	Married	MSs Adult Nursing	14	MSc Nursing
29	FGDP24	Female	35	Orthodox	Married	BSc Nursing	13	BSc Nursing
30	IDI6	Female	36	Protestant	Married	MSs Community Psychiatry	16	MSs Community Psychiatry
31	IDI7	Male	31	Protestant	Single	BSc Nurse	8	BSc Clinical Nursing
32	IDI8	Male	30	Protestant	Single	BSc IESO	10	BSc IESO
33	IDI9	Male	34	Protestant	Married	Master’s of Public Health	8	Public health and quality control
34	IDI10	Male	41	Protestant	Married	MSc in adult Nurse	16	Adult nurse

Note: IDI, in-depth interviewee, FGDP, Participants of Focus Group Discussant, IESO, Integrated Emergency Surgical Officers, PH, Public Health

### Participants’ conceptualization of common occupational mental health symptoms

Before, exploring participants’ perceived causal link between inadequate WASH and common mental symptoms, we first explored participants’ awareness of, vulnerability to, subjective experiences of, and perceived causal link between inadequate WASH as work stressors and perceived controllability of common mental symptoms. The participants’ conceptualisation of common mental health symptoms was assessed using self-identification as having common mental health symptoms (SICMS) [12] frameworks.

Accordingly, many participants had low symptom recognition, high perceived vulnerability to, high subjective experience of, and high perceived causal link between inadequate WASH as work-related stressors at the points of care areas. Participants also had a low perceived ability to control and prevent occupational stress believing it as if it was a normal part of work life. Similarly, participants had low cardinal symptom identification status and high perceived vulnerability to common mental symptoms. Many healthcare workers also had a high perceived link between inadequate WASH-related stressors and depressive and anxiety symptoms, but low perceived ability to identify causal pathways, and perceived difficulty in preventing and controlling both symptoms. Furthermore, the majority of participants had low subjective experience with both occupational depression and anxiety. Key findings and sample quotes for each common mental symptom discussed are summarised in Table 2.

**Table 2:** Health workers’ awareness about, vulnerability to, subjective experiences of, belief in the causal link between work stressors and CMSs, and perceived controllability of common mental symptoms.

Health workers conceptualization elements of common mental health symptoms	Key findings	Sample participant’s quotes
Occupational stress		
Awareness/symptom recognition	Only four out of 34 participants fully comprehend occupational stress symptoms(low symptom awareness)	*“Emm*, *Let alone defining ‘occupational stress’; I am afraid of defining ‘stress despite a daily term we [health workers] use*. *[…] I think stress means overwhelming the mind with tasks*. *It occurs when resources are not fulfilled in the workplace to meet work goals*. *[…] I think stress symptoms are ambiguous and interconnected with other mental illnesses*. *[…]”* ***(A male*, *34 years old*, *BSc in Nursing)***
Vulnerability to occupational stress	A majority believed they could encounter anxiety and perceived that the disease may impact them as any other form of chronic mental symptoms.	*“Yes*, *despite having inadequate information to identify symptoms*, *I strongly believe that I may have experienced stress at various points in my life due to work*. *[…]Because of my workload*, *I suffer from persistent back pain*. *I am also worried about how my health will be affected in the future*. *Along with the stress*, *additional health issues may develop in the future*.*”* ***(Male*, *36 years old BSc Nurse & BSc in public health)***
Subjective lived experiences	Almost all health workers subjectively perceived that they had the condition in their professional lives.	*“I realized I was stressed*, *but I wasn’t fully aware of how severe it was*, *how long it had lasted or the exact moments that had caused it*. *[…] I did not consult any mental health professionals*. *I had experienced symptoms like physical*, *mental*, *and emotional*. *[…] However*, *how did I know if it was chronic stress or not*? *How could I identify whether my symptoms were linked to specific work-related stressors*?*”* ***(Male*, *30 years old*, *BSc IESO)***
Believes of causal or risk factors for work-related stressors and occupational stress	Most health workers believe that work stressors cause them to have increased levels of stress.	*“For me*, *[…] it is difficult to describe pathophysiological mechanisms that associate work stressors with specific stress symptoms*. *Knowing the duration of symptoms experienced also adds complexity*. *[…] lack of clinical guidelines in our health facilities could result in the confusion surrounding multiple causes of stress*. *[…] I can’t be certain if a particular work stressor is linked or linked to other life stressors*. *As a result*, *it is difficult to draw any clear conclusions about a causal link*.*”* ***(Male*, *36 years old*, *BSc in Clinical Pharmacy)***
Perceived controllability and prevention	Many of the participants faced difficulty in differential self-diagnosis for occupational stress as a result they had a perceived view of the inability to control	*“As previously discussed*, *workload was the main reason for high-level stress*. *However*, *how can it be prevented*? *If I seek counselling for this issue*, *what steps can I take to improve the situation*? *Without institutional prevention strategies*, *the situation is unlikely to improve*. *Counselling or any behavioural change interventions could help*, *but changing the situations will require broader systemic change*. *I do not know how we could change*.*”* ***(Female*, *42 years old*, *MSc Adult Nursing)***
Occupational anxiety/job anxiety		
Awareness/symptom identification	low awareness of cardinal symptoms of occupational depression	*“Honestly*, *I don’t understand ‘job anxiety’ or ‘occupational anxiety’*. *I can’t even mention the symptoms of job anxiety*, *nor can I differentiate it from depression or stress […] It is hard to distinguish between the symptoms of stress*, *anxiety*, *and depression that I experience at work*. *[…] Of course*, *guess we must be very aware of symptoms of this mental phenomena*.*”* ***(Male*, *31 years old*, *BSc Nurse)***
Vulnerability to occupational anxiety	The majority believed they could encounter anxiety.	*“[…] I believe anxiety is because by frequent exposure to bad work-related incidents*. *Of course*, *identifying which work stressors are contributing to it is difficult*. *However*, *I am afraid of developing the disease*. *Sometimes*, *my wife overhears calls me talking loudly about work-related issues*, *and calls*, *concerned*. *I think that could be a yellow flag for my fear of getting the disease*.*”* ***(Male*, *55 years old*, *BSc Nurse)***
Subjective lived experiences	Few participants suspected that could have had this symptom.	*“I don’t believe I experienced anxiety symptoms at work*. *However*, *I know two health workers; a midwife and a clinical nurse*, *who have been diagnosed with this condition*. *[…] However*, *I am not sure if their cases are linked to work factors*.*”* ***(Male*, *36 years old*, *BSc pharmacy)***
Believes of causal or risk factors for work-related stressors and occupational anxiety	Many health workers believe that work-related stressors can cause or increase the risk of anxiety.	*“I am not aware of the exact role that work-related stressors play in anxiety*. *[…] Anxiety can have diverse causes and symptoms*. *Many of the stressors we previously discussed make me feel nervous*, *uneasy*, *and fearful about negative medical outcomes for my patients*. *[…] I believe that these factors truly lead to anxiety*.*”* ***(Male*, *33 years old*, *MD)***
Perceived controllability and prevention	many health workers believed that ‘anxiety’ in general including perceived ‘occupational anxiety” could be difficult to control or prevent	*“Most of us lack experience of consulting a mental health professional*. *One reason may be the stigma surrounding mental health issues*, *such as the fear that a healthcare professional could suffer from depression or anxiety […] […] healthcare workers might think that mental health symptoms like anxiety are difficult to control or overcome easily*. *We might also think that our patients will not believe in us to receive services”****(Male*, *31 years old*, *BSc Nurse)***
Occupational depression		
Awareness/symptom identification	Most health workers had low awareness of the cardinal symptoms of occupational depression.	*“I associate anxiety with feelings of being ‘depressed’ or having ‘mood swings’*. *Even without considering work-ascribed forms*, *I struggle to understand my feelings when I get depressed*. *My college course work covered the topic of depression*, *but it didn’t prepare me for how I would feel*. *[…]At this moment*, *I’m thinking about my work*, *and feel as though have always felt this way***.*” (Male*, *29 years old*, *BSc Laboratory)***
Vulnerability to occupational depression	Most health workers fear that they will acquire occupational depression.	*“Yes*! *I believe these mental health disorders can be linked to workplace stressors*. *[…]I not only fear experiencing symptoms*, *but I also worry that I may develop a depressive disorder later in my life*. *I can’t recall my previous experiences with depressive symptoms*. *[…] And*, *I have no idea how to avoid extreme challenges since many stressors are beyond my control*.*”* ***(Male*, *28 years old*, *BSc Midwife)***
Subjective lived experiences	Most participants did not believe that they had depression.	*“Yes*! *I don’t believe I’ve experienced symptoms of depression*. *However*, *a colleague confided in me that he has experienced depressive symptoms*, *and had feelings of sadness*. *[…]*. *He asked me to keep this information confidential and mentioned that he was advised to take a break*.*”* ***(Male*, *29 years old*, *BSc Radiology Nurse)***
Believes of causal or risk factors for work-related stressors and occupational depression	Many health workers believe that work stressors increase depressive symptoms.	*“[…] I believe they have a link*. *However*, *it’s challenging to describe the precise pathophysiological pathways that link work stressors with specific symptoms of depression*. *Recalling the difficulty of the duration of symptoms further complicates this understanding*. *[…]*, *[…] I am uncertain if a particular work stressor is directly linked to depression or if it is caused by other life stressors*. *Therefore*, *it would be difficult to establish a clear causal relationship*.*”* ***(Male*, *36 years old*, *BSc in Clinical Pharmacy)***
Perceived controllability and prevention	Many health workers were afraid of the inability to control or prevent work factors.	“[…] I don’t think that all my colleagues are free from depressive symptoms. They were simply afraid of coming to this psychiatry room. […] They often do not believe […] depression and other mental illnesses as anyone’s health concern. […] If you look closely, intellectuals, including health professionals perceive mental illnesses as manifestations of evil spirits.” ***(Female 36 years old*, *MSc Community Psychiatry)***

Despite there being some paradoxical findings between low symptom identification skills and high perceived vulnerability to common mental symptoms, health workers perceived or appraised that their work-related stressors, including inadequate WASH, went beyond their control capacities, leading to the belief that work-related stressors, including WAHS, caused them to experience increased symptoms of common mental illnesses.

### Emerged themes

Four themes emerged from coding segmented data and categorising them based on the constructs of two theoretical models relevant to the study questions: ‘Exposure to inadequate WASH and perceived link with common mental symptoms’, ‘Perceived impact of inadequate WASH on professional quality of life’, ‘Experience of coping with inadequate WASH’, and ‘Barriers to reducing inadequate WASH as stressors to mitigate mental health impact’.

**Fig 1:**
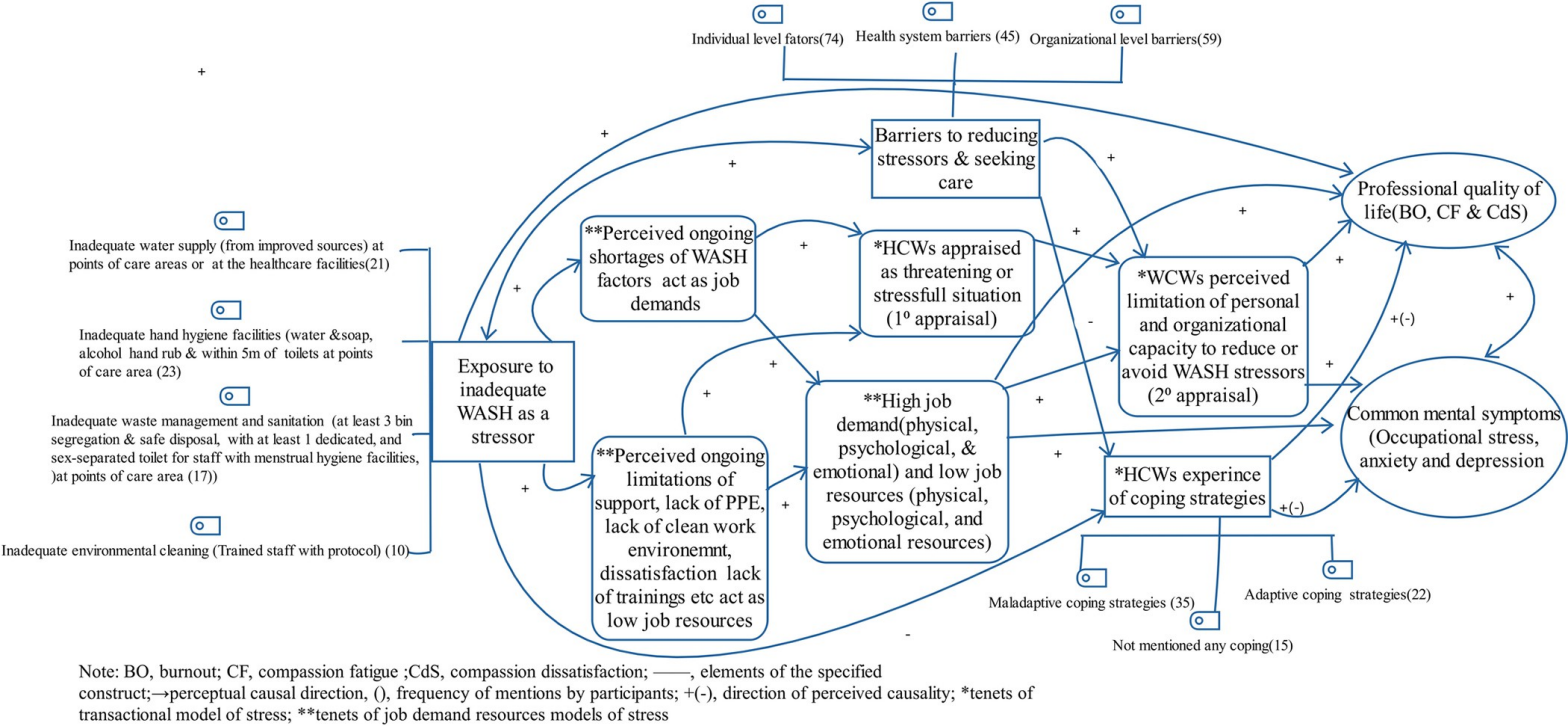
Perceptual link between inadequate WASH with common mental symptoms, and professional quality of life in health workers, February 2023.

Fig 1 illustrates the relationships between the themes that emerged from the data. The causal pathway is drawn from the health workers’ perceptions or beliefs expressed during the interviews and the discussion based on the guiding theoretical model of psychological stress: TMS [4] and JDR [5]. As observed from the perceptual causal directions in Fig 1, one can understand that health workers’ exposure to inadequate WASH and other important factors mentioned in each theme may have at least the following interrelationships: (1) inadequate WASH is perceived to increase the risk of CMSs through negatively affecting coping strategies(personal psychological and physical resources), professional quality of life (burnout, compassion fatigue, compassion satisfaction), and by exacerbating the negative impact of barriers; (2) inadequate WASH is also perceived to negatively influence coping strategies (e.g., participants felt that WASH issues were being individually unaddressed), and barriers at all levels can also cause inadequate WASH to be a persistent stressor; (3) poor coping strategies for healthcare workers, barriers at all levels and professional quality of life (e.g., burnout and compassion fatigue) together with inadequate WASH increase the risk of CMSs.

### Theme 1: Exposure to inadequate WASH and perceived links with common mental symptoms

**Fig 2:**
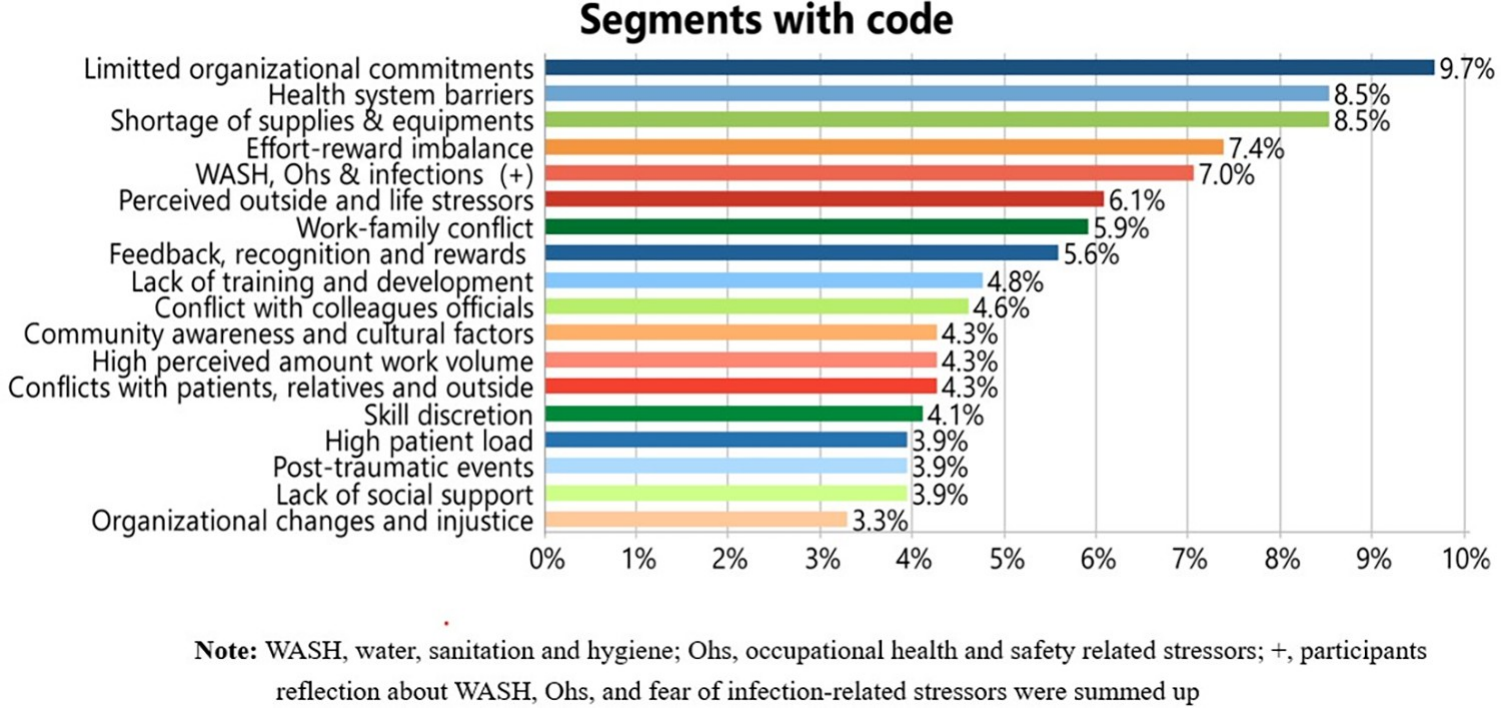
Frequency of mentions of common mental symptoms as perceived stressors among health workers in the Central Ethiopia Region, February 2023.

We explored how health workers comprehend symptoms of stress, anxiety, and depression. We then explored how they associate inadequate WASH as a workplace stressor with each symptom after asking participants about the cardinal symptoms of each mental illness. Although not all health workers listed each symptom precisely, their subjective reflections were considered perceptual causes through the interpretive coding strategy of segmented data. According to the participants, inadequate WASH was ranked as the fifth most mentioned work-related stressor in the points of care areas, as illustrated in Fig 2, which summarises the code frequency of the segmented data.

Most participants perceived shortages of functional hand hygiene facilities, a shortage of continuous clean water supplies for sinks and nonfunctional sinks, a lack of soap, nonfunctional or unavailable washing facilities at 5-meter toilets, and alcohol-based hand rub at points of care areas. Additionally, many participants reported experiencing a shortage of water for cleaning floors, medical equipment, and other sanitation and hygiene-related tasks in the hospital. One participant mentioned:

*“[…] Most sinks lack water due to interruptions in supply. […] Soapis often unavailable. […] The only sinks with water are in the surgery and intensive care units. I always fear the risk of infection. I’m also concerned about how patients and visitors perceive us. How can I address this problem? I believe that this persistent worrying contributes to a depressed mood.”* ***(Female, 37 years old, MSc in Community Psychiatry***)

Most participants perceived inadequate basic waste management services, including the unavailability of at least three bins for segregation, unsafe segregation practices, and improper disposal of medical waste. These issues caused health workers to fear for their safety as well as that of their patients, and visitors at points of care. Many believed that these factors were among the most persistent stressors in these settings, potentially increasing the development of symptoms related to stress, anxiety, and depression. One of the participants reflected on these problems:

*“Sometimes, waste bins are relocated to another ward, leaving our ward without one. […] Additionally, staff and patients toilets are not separated. […] It is always difficult to keep my hygiene as I desire.[…] I believe fear of infection due to such limitations contributes to persistent stress, a depressed mood, and excessive worry”. (Male, 35 years old, BSc in Nursing*)

The majority of health workers reported substandard implementation of infection prevention control (IPC) for both cleaners and healthcare staff. Almost all participants indicated that they had received IPC training; however, practice-related gaps persisted. One participant stated the issues and the perceived link between environmental cleaning at points of care, and mental health symptoms.

*“Cleaners often lack awareness of infection prevention and control (IPC) protocols. They [cleaners] believe that cleaning once a day is adequate, which increases our workload and raises concerns about infections and negative care outcomes. I worry about infections affecting myself, my patients, and my family. Yea, such persistent worry can cause mental strain, tension, depression, and demotivation.”* **(Male, 41 years old, MSc in Adult Nursing)**

Many health workers expressed concerns that inadequate WASH challenged their ability to manage job demands in various ways and decreased their hope of using their personal coping and resilience strategies as if the problems could not be resolved at the individual level. One participant stated that the interaction between inadequate WASH and other stressors, particularly challenges related to infection prevention and control activities, exacerbated their psychological difficulties.

*“[…] Hygiene and sanitation are persistent challenges here [at the hospital]. […] Compliance with infection prevention and control practices is difficult. We have no separate water source for the hospital. We get water from the town. Only a few hospital units, such as the surgery wards, intensive care units, and delivery wards receive water from reservoirs.[…] Imagine how this can hinder us from meeting our goals, creating discomfort, and negatively impacting the community’s perception of us [healthcare workers], causing distress for patients and visitors. I think this could also make us fail to control and cope with other stressors.”* ***(Male, 34 years old, BSc in Community Psychiatry)***

### Theme 2: Perceived impact of inadequate WASH on professional quality of life

Under this theme, we explored the perceived impact of exposure to inadequate WASH on professional quality of life (PQoL) using two key components of PQoL [27]: compassion fatigue, and burnout feelings, as well as compassion satisfaction. The components of PQoL, particularly burnout and compassion fatigue, may also contribute to increased distress, anxiety, and depressive symptoms, as shown in Fig 2, which describes the relationships among the themes. Similar to the challenges in identifying symptoms of common mental illnesses, many health workers struggled to identify the independent contributions of inadequate WASH This included difficulties in recalling each episode of symptoms concerning duration and intensity, as well as establishing independent links with any components of WASH. Nevertheless, many health workers believe that inadequate WASH, in conjunction with other work stressors increases the risk of decreased compassion satisfaction and increased burnout. A participant summarized the impact of insufficient WASH and the imbalance between high effort and low reward (another stressor) as follows:

*"[…] I switched from laboratory to the nursing department 2 years ago, but the challenges are almost the same. Forget about better pay, I do not even have a clean workplace. No matter how much I scrub the floor never feels clean. Sometimes, the disinfectants, and cleaners we need are not available. I am always afraid of being infected due to spill overs of urine, blood, and bodily fluids. As you can sense at this moment, it smells unbearable. I worry every day about contamination. […] Most shifts, I’m the one cleaning, even though I’m supposed to be a nurse. I can’t even list all the problems. I can’t list all the problems. […] I’m exhausted, frustrated, and bored. I need to get out—before I lose myself to this job."* ***(Male, 32 years old, BSc Nurse)***

Many participants also worried about the medical outcomes of their patients, but there were many persistent challenges in WASH near the points of care. A participant, for example, stated that he was emotionally drained and physically exhausted due to prolonged exposure to persistent inadequate WASH and other stressors at the points of care.

“*As a doctor, I can’t imagine the pressure of working or performing a surgical procedure in an environment lacking proper hygiene and cleanliness. The use of stored water for cleaning, occasional lack of disinfectants, inadequate handwashing facilities, and improper waste management greatly concern me. What terrifies me even more is thinking about what it would feel like as a patient. Would I feel safe, or would I be gripped by fear knowing the risks?”* **(Male, 33 years old, MD)**

### Theme 3: Coping experience with inadequate WASH

This theme explored the strategies health workers employed to cope with the inadequate WASH component they raised during the interviews and discussion. We categorized coping strategies into two groups according to the TMS theoretical framework [4]: adaptive (problem-solving and/or positive emotion coping strategies), and maladaptive (negative action and/or negative emotion thought) strategies. Another classification for summarizing the findings was for those who either did not recall any coping strategies or had no planned coping strategies. Accordingly, many health workers felt that addressing WASH issues necessarily requires intervention from higher-level bodies or authorities such as sectorial officials as individual efforts had a limited impact. Additionally, nearly equivalent numbers of participants either did not recall or did not have planned coping strategies with inadequate WASH. One participant shared insight into how his attempts to solve inadequate WASH problems at the individual level had been unsuccessful, illustrating the challenges faced by many health workers in this regard.

*“[…] We remind hospital officials to keep water tankers filled as a reserve. I usually rely on alcohol-based hand rubs for hand hygiene. But, sometimes they aren’t available. What should I do, for example, in case of a shortage of water? In the past, I did not take any steps to prepare for it. Can I or my colleagues do anything about it?” (* ***Male, 28 years old, BSc Midwife)***

Study participants believed that they could reduce the negative consequences of inadequate WASH on common mental symptoms, mainly by avoiding the problem. However, only a few participants felt that they could have an impact through emotional preparation, such as seeking psychological support or other emotional copping strategies. One participant, a general practitioner, advised health workers should emotionally prepare themselves to accept these problems and seek support from others.

*“I think we can think of emotional self-management by considering acceptance as a solution alongside personal coping efforts and support from hospital officials. However, most of us tend to see problems like inadequate WASH and other stressors as overwhelming threats that we feel powerless to address.”* ***(Male, 33 years old, MD)***

### Theme 4: Barriers to reducing inadequate WASH as a stressor

In this theme, we explored health workers’ experiences with the barriers they faced to improving coping with inadequate WASH. Many healthcare workers frequently encounter barriers to improving at the individual level, such as inconsistent waste segregation practices (improper use of labelled bins, relocation of waste bins, shortage of labelled waste bins), and a tendency to only rely on higher authorities to solve these issues. Additionally, there is often a lack of organizational commitment to maintaining proper WASH infrastructure, and other system-level gaps.

Although healthcare workers acknowledge the impact of inadequate WASH on mental health outcomes, many of them primarily look to external bodies for solutions. One of the participants expressed concern about implementing IPC measures mentioning one of the most important components, segregation practices.

*“We face enormous challenges with waste segregation in the care areas. […] Even with IPC training, implementation is still lacking. If we worry about infection, we must ensure strict adherence to proper protocols.”* ***(Male, 41-year-old, MSc Adult Nurse)***

Similarly, participants raised organizational-level barriers. These included a lack of commitment to hospital WASH initiatives and insufficient mental health-related training for coping with such problems. One participant described the commitment challenge for the hospital management.

“*During the COVID-19 pandemic, there were adequate portable alcohol hand sanitisers in the hospital. However, in the aftermath, we faced a troubling scarcity of these essential items, highlighting significant shortcomings in the administration’s ability to maintain critical supplies.”* ***(Female, 37 years old, MPH&Nurse)***

Participants raised many system-level barriers that require collaborative efforts between the health system, water supply, sewerage, and municipal waste management bureaus to alleviate inadequate WASH.

“*We have no separate source of water for the hospital. The city’s water supply and sewerage services must work together with the health system to ensure a continuous supply. […] Currently, less than 10% of the points of care points receive continuous water, as it is delivered in rotation, just like in the surrounding community. However, this kind of limited access is unacceptable for healthcare facilities.”* (**Male, 55 years old, BSc Nurse)**

## Discussion

Our study explored health workers’ perceptions of the causal links between inadequate WASH and common mental symptoms, as well as their views on the impact of inadequate WASH on professional quality of life. We also examined their experiences of coping with inadequate WASH and identified barriers to addressing these issues as sources of distress. In the process of exploration of perceptual causation, perceptions and feelings or actual experiences of health workers or their colleagues, with common mental symptoms were first explored, and then we examined how these perceptions could be linked to inadequate WASH. As displayed in Fig 1, health workers subjectively or raised one or more of the components of WASH such as inadequate water supply from improved sources at points of care areas or the healthcare facilities, hand hygiene facilities (water &soap, alcohol hand rub, and within 5m of toilets at points of care area), waste management and sanitation (at least 3 bin segregation followed by safe disposal, with at least 1 dedicated sex-separated toilet for the staff with menstrual hygiene facilities) at points of care area, and environmental cleaning (trained staff with protocol. Then, individual healthcare workers were asked to link specific inadequate WASH components with symptoms of common mental illnesses, and symptoms of professional quality of life.

Individual perceptions of WASH-related stressors and feelings associated with common mental illnesses, and symptoms of poor professional quality of life could provide insight into the causal links and inform the development of stress management interventions at the individual, organizational, and system-levels. Understanding how health workers perceive, interpret, and appraise inadequate WASH as a stressor is essential to assess the link between the stressor and its somatic psychological manifestations regardless of its severity. This understanding can serve as a foundation for mitigating or reducing inadequate WASH-related stressors or for establishing adaptation measures, ultimately offering valuable insights for policymakers, implementers, and health workers.

Despite the complexity of tracking invariability of difference-making and causation, counterfactuals, regularity, and necessity [28], a perceptual causal understanding among health workers provides insights into the risk that inadequate WASH increases the likelihood of reporting mental symptoms. Although health workers self-identify very little with having common mental symptoms, our findings highlighted that inadequate WASH led them to experience symptoms of distress, anxiety, and depression. Many of their perceptual links were related to fears of infection (both for themselves, patients, and visitors), fears of reduced performance, and worries about medical errors (e.g., poor outcomes for the patients they care for). Health workers’ accounts of fears are substantiated by risks associated with poor WASH and failure to maintain infection prevention control, such as hospital-acquired infections (HAIs) [29], antimicrobial-resistant infections (AMRIs) [30], and needle-stick injuries [31]. Furthermore, fears related to psychological safety may arise from the perception that managers are unsupportive and unaware of health workers’ safety concerns, which could further increase the risk of severe mental symptoms among health workers, as documented in another study [32]. In all scenarios, health workers may perceive the situation as threatening and feel unable to cope with it. They may continue to fear the persistent consequences of inadequate WASH, leading to mental strain reactions according to the TMS [4] mentioned earlier. Additionally, health workers’ ongoing persistent exposure to inadequate WASH and their perceived inability to control the situation due to resource constraints (e.g., psychological, material, and financial) may contribute to mental strain, in line with the JDR [5]. This can extend to other mental health outcomes, including anxiety and depression [33].

In our search, we could not find either qualitative or quantitative studies demonstrating the perceptual link between inadequate WASH and common mental health symptoms in healthcare populations. However, despite differences in population, setting, and study design differences, a meta-analysis supported the perception that inadequate sanitation and water insecurity increased the odds of having common mental disorders, including mental distress, anxiety, and depression. Of the studies included in the review, three were from Ethiopia: one study indicated that water insecurity heightened the risk of psychological distress, including stress, anxiety, and depression, in the general population [34] and especially in women [35]. Another study from northeastern Ethiopia found that water insecurity also increased the risk of psychological distress, including mental distress, depression, and anxiety [36]. Similarly, another systematic review showed a positive correlation between hand hygiene, both depression and anxiety [37] in pandemic settings, and the use of unimproved water sources and poor sanitation and major depressive disorder [38]. Despite variations in population, our study may support participants’ perceptions of these inadequate WASH components, as not all studies address common mental distress from cause-specific perspectives.

The frequency with which health workers mention inadequate WASH-related stressors as Fig 1 suggests a heightened likelihood of perceiving inadequate WASH as a threat. This perception may contribute to the reporting of mental health symptoms among healthcare workers. In other words, the subjective experience of health workers with serious inadequate WASH as high job demands and low control may lead to manifestations of mental health symptoms. Given that WASH-related infrastructures require significant attention and time, the probability of cumulative chronic exposure to inadequate WASH as a stressor over time is elevated, contributing to disorders such as chronic stress, anxiety, and depression. Therefore, our findings suggest the need for adaptive coping mechanisms in response to inadequate WASH, in addition to enhancing access to safe basic water, environmental cleaning, waste management, and hygiene services at points of care.

Providing technical training for health workers tailored to coping with inadequate WASH in care areas of healthcare facilities facing water shortages and related hygiene supply issues could yield promising outcomes for maintaining the well-being of health workers. To achieve sustainable solutions, there should be a special emphasis on increasing basic WASH services in healthcare facilities, particularly in low-income countries such as Ethiopia, which face challenges from global inflation in covering the costs of constructing and maintaining sanitation and health facilities. Additionally, supporting improved access to technological and technical skills will help mitigate the impact of climate change that may pose water supply shortages to healthcare facilities.

Similarly, our study showed that inadequate WASH caused health workers to experience mental exhaustion, including feelings of hatred toward their work and intentions to leave (burnout, a key component of the PQoL), as well as feelings of fear of conducting surgical procedures in unhygienic conditions (compassion fatigue) due to water, and hygiene shortages. This may deplete their ability to cope with WASH-related stressors and other and other workplace stressors. We acknowledge that our findings may lack strong evidence as health workers had difficulty articulating how each component of the WASH is linked to symptoms of compassion fatigue during our interviews and discussions. Additionally, there are no studies specifically linking WASH and symptoms of PQoL within the healthcare population in the Ethiopian context for comparison. Adequate WASH could serve as a potential resource for helping health workers prevent burnout and compassion fatigue, as well as reduce workload, and high job demands. Studies also indicate that workload and insufficient resources in the workplace contribute to increased burnout, decreased life satisfaction [39], heightened emotional exhaustion and decreased job satisfaction, all of which lead to compassion fatigue [40, 41]. Although the issue of inadequate WASH supports this conclusion, we recommend further studies to explore the impact of various components of inadequate WASH on each element of professional quality of life. This could have both individual health workers’ mental health implications and the quality of healthcare as pertinent strategies of the healthcare system of Ethiopia [42].

We could not find studies specifically measuring coping strategies for inadequate WASH as stressors to compare with the coping strategies employed by health workers when dealing with inadequate WASH. COVID-19-era studies from Italy have shown that physicians use problem-based coping strategies, such as actively addressing the challenges and demands of patient care, rather than other coping strategies, such as emotion-based strategies, avoidance or maladaptive coping strategies [43]. Another study showed that those healthcare workers who used problem-based adaptive strategies such as reducing or avoiding the stressor, seeking social support, and active hands-on approaches towards the problem, and who had positive psychological conditions had positive emotional health compared with health workers who used emotion-based negative coping such as avoidance/escaping [44]. This study aligns with an earlier study by Lazarus and Folkman, which showed that relying on negative emotional coping strategies without addressing the root cause leads to increased stress, resulting in chronic stress, depressive symptoms, and fatigue [17]. However, the lack of planned coping strategies or the use of negative coping strategies, as observed in our findings, raises the risk of experiencing common mental health symptoms. Once health workers re cognise that they are facing problems or stressors, in this case, inadequate WASH, a response is essential, and it would be beneficial to develop important individual coping strategies.

The type of coping strategy employed is highly dependent on the stressful event and context. The coping strategies planned for persistent work stressors, such as inadequate WASH may differ from those employed for other acute adversities. Even the earliest individual coping strategies suggested by Lazarus and Folkman [17] and later developed by Dewe [18] include rational task-oriented behaviours, emotional release, utilizing home resources, recovery and preparation, postponing action through distracting attention, and passive attempts to tolerate effects. These strategies need to be supplemented with new context and technology-oriented approaches. However, perceiving inadequate WASH as a nonmodifiable risk factor due to heavy investment from organizational and system-level entities should be minimized, and panning for individual coping strategies remains crucial. Evidence shows that individuals possess personal resources that enable them to proactively cope with job-related stressors [45]. Hence, once health workers identify inadequate WASH as a significant factor contributing to negative mental feelings, they need to consult and innovate approaches [46] to mitigate its effects.

Last, the study explored various barriers to coping with work-related stressors and seeking support from mental health services for persistent mental health symptoms. These barriers were contextual, and identified at the individual, organizational, and health system levels observed from the frequency with which contextual layers of stressors were mentioned, inadequate WASH elements as stressors are among the most commonly cited workplace stressors. These stressors may be further intensified by other workplace environment stressors such as limited organisational support, conflicts with colleagues and management, a lack of professional training and development opportunities health sector-level factors and other system-level factors. Addressing these systemic issues, such as improving water supply to healthcare facilities and ensuring a steady supply of hygiene products (detergents, soaps, s sanitisers, alcohol-based hand rubs), could help reduce the mental health impact on healthcare workers by alleviating fears related to physical health risks and improving their work environment. All these could reduce fear of physical health threats and bad emotional feelings at work due to poor aesthetic conditions and increase the daily functioning of health workers to better medical outcomes through providing quality care.

Therefore, our findings suggest a need for training health workers in emotional and physical problem-solving skills, as well as practices related to WASH (such as waste management, environmental cleaning, and ensuring hand hygiene facilities at the points of care), along with the implementation of other innovative strategies. Similarly, our findings highlighted a need for organizational and system-level interventions to ensure a clean water supply for healthcare facilities and establish alternative clean reservoirs through intersectoral collaboration with water services, and infrastructure innovation services for effective water use. Testing effective individual interventions [47, 48] for health workers, along with WASH-related interventions tailored for low-income countries [49, 50], may be needed to scale up the most effective strategies to fit different contexts. Collaboration and integration of interventions aimed at reducing organizational and system-level barriers in the workplace may also be essential.

### Trustworthiness and limitations of the study

Exploration of common mental symptoms such as stress, anxiety, or depressive symptoms along with their effects followed by how participants link each component of WASH to these symptoms as causes or risks, would enhance our understanding of perceptual causation. Identifying symptoms (stress, anxiety, or depression), and assessing the duration and specific nature of these symptoms associated with each component of inadequate WASH can help reduce recall bias. Utilizing a theoretical framework may support a causation explanation of how inadequate WASH contribute to common mental symptoms. We ensured the trustworthiness of our study through various strategies. Member checking was used to validate our interpretations by summarizing narratives for participants’ confirmation, thereby enhancing credibility. We documented every step of the research process including data collection, analysis, and interpretation to ensure dependability, employing explicit, and systematic coding based on theoretical frameworks to maintain transparency and allow for replication by future researchers. The inability of professionals to review coding samples and the discussions with researchers contribute to confirmability. We provided a detailed description of the study context, participants, and research methods, with findings for each theme supported by participants’ quotes to enhance transferability.

However, despite the conceptualization and expectations surrounding mental health symptoms, health workers found it challenging to recall specific mental symptoms (stress, anxiety, or depression) and specific stressors (s). This may complicate the verifications of whether changes in feelings were influenced by specific stressors and hinder the assessment of the variability of exposure to those stressors [28]. Difficulties in recalling specific stressors, the duration of exposure, and their interaction with a specific mental health symptom or multiple symptoms whether singly or in combination with other stressors would limit the ability to provide a sufficient counterfactual, regularity, necessity, and invariability of causation [28]. This suggests a need for additional mixed study designs [28] and longitudinal qualitative [10] research, because, the causal relationship between psychosocial determinants such as inadequate WASH and mental health symptoms is complex. Similarly, determining which coping strategies corresponded with a specific stressor(s) and/or symptoms of common mental disorders was difficult due to health workers’ recall bias, despite efforts to elicit coping strategies during our interviews and discussions.

## Conclusions

Healthcare workers perceived inadequate WASH components caused them to manifest symptoms of common mental disorders and negatively affect their PQoL. They also perceived inadequate WASH components as threats believing it as non-modifiable risk. This implies that healthcare workers may not be motivated to take any individual measures as a result of perceiving the challenge could only be solved at the organizational or system level. Ultimately, failure to cope may exacerbate feelings of common mental symptoms and reduce professional quality of life. They faced multiple barriers at individual, organizational, and health system levels, which hindered their ability to manage work-related stress and seek mental health support.

Due to perceptual and recall biases, differentiating whether specific inadequate components of WASH as stressors, with other workplace stressors with common mental health symptoms will be difficult due to limitations to correctly recording the duration, and magnitude of exposure to those stressors, and other confounders limit in providing a counterfactual, regularity, necessity, and invariability of causation. Therefore, we recommend additional mixed (cohort studies with longitudinal qualitative study) designs to determine whether inadequate WASH increases the risk of developing or exacerbating mental health symptoms.

### Policy implications and recommendations

With limitations specified before, our findings still highlighted the need to integrate WASH and mental health services for healthcare workers, which could involve ranging from establishing individual-level interventions and promoting collaborative efforts among hospital administration, health systems, water and sewerage services, and various national and international organizations. The healthcare policies should also invest in better water supply, sanitation, environmental cleaning, and waste management at the points of care and healthcare facilities in general. Mental health support systems and strategies for with coping WASH-related stressors including counselling and stress management as workplace wellness packages, should be tailored for healthcare workers. Additionally, ongoing education on WASH protocols and workplace well-being monitoring approaches will enhance both WASH standards and mental health outcomes, ultimately improving healthcare workers’ professional quality of life.

## Supporting information

S1 File Cardinal symptoms of generalized anxiety and depressive symptoms for guiding perceived link between the WASH stressors, and common mental symptoms among health workers, February, 2023. (PDF)

S2 File Standards for reporting qualitative research (SRQR) for the study of perceived link between WASH related stressors, and common mental symptoms among health workers, 2023. (PDF)
