# Supplementary material for: Perceptual link between inadequate water, sanitation, and hygiene (WASH) stressors and common mental symptoms in Ethiopian health workers: A qualitative study
Source: PLoS One. 2025 Jan 10;20(1):e0314170. doi: 10.1371/journal.pone.0314170 (PMC11723618; doi:10.1371/journal.pone.0314170)
Supplement: S1 File — (PDF) [file pone.0314170.s001.pdf]

S1 file: Cardinal symptoms of generalized anxiety and depressive symptoms for guiding perceived link between the WASH stressors, and common mental symptoms among health workers, February, 2023.

1. **Cardinal symptoms of Anxiety**

Common symptoms associated with general anxiety disorders were assessed based on (DSM-5). If participants could recall each of them, their own subjective perceived definition of anxiety was accepted by the interviewer.

Step 1: Health workers asked how they define “anxiety” based on (DSM-5) considering the persistent presence of specific symptoms, and if they couldn’t recall time subjective definition was accepted.

Step 2: Then, ask whether the cardinal symptoms are associated with the mentioned work stressors, including WASH-related stressors if they contributed to the specific symptoms. For instance, if a health worker reports a cardinal symptom like 'excessive worry,' interviewers should explore how the worker subjectively links this to specific job stressors. Here are the cardinal symptoms of depression based on DSM-5 criteria:

- **Excessive worry:** Constant, uncontrollable worry about a wide range of events or activities and difficulty controlling the worry.
- **Restlessness or feeling on edge:** A sense of restlessness or feeling easily fatigued and being on edge or feeling irritable.
- **Muscle tension**: Physical symptoms such as muscle tension, aches, soreness, and restlessness or feeling keyed up.
- **Sleep disturbances:** Difficulty falling asleep or staying asleep and restless, unsatisfying sleep.
- **Difficulty concentrating:** Feeling mentally tense or on the edge and difficulty concentrating or finding that the mind goes blank.
- **Irrational fears:** experiencing irrational fears or excessive worry about specific objects, situations, or activities (in the case of specific phobias) and panic attacks in response to the feared object or situation.
- **Avoidance behaviours:** avoidance of certain places, situations, or activities due to intense fear or anxiety and significant disruption to daily life and functioning.
- **Physical symptoms:** Various physical symptoms such as sweating, trembling, shortness of breath, palpitations, or a feeling of choking (in the case of panic disorder).

NB: Probing continued for those health workers who could provide at least one symptom, otherwise stopped and proceeded to the subjective link between the work stressors including WASH-related stressors and anxiety.

2. **Cardinal Symptoms of depression**

Health workers' perceived and actual knowledge of depressive symptoms was assessed against DSM-5 criteria for diagnosing depressive disorder.

- **Step 1.** Health workers asked how they define "depression" considering the consistent presence of specific symptoms over at least two weeks, and if they couldn't recall time subjective definition was accepted.
- **Step 2.** Then, ask whether the cardinal symptoms are associated with the mentioned work stressors, including WASH-related factors in a similar way the Occupational Depression Inventory assess how the symptoms are linked to their work. For instance, if a health worker reports a cardinal symptom like 'depressed mood,' interviewers should explore how the worker subjectively links this to specific job stressors. Here are the cardinal symptoms of depression based on DSM-5 criteria:

**Cardinal symptoms**	Asking the general definition or meanings of depression if they can mention them through probing, and listening to whether health workers mention cardinal symptoms of depression based on DSM-5	Asking or exploring how to link work stressors including WASH-related stressors with the health worker's subjective experiences if they believe that the two are linked to each other.
Depressed mood	A pervasive feeling of sadness or emptiness that is present most of the day, nearly every day.	Believe that your work stressor did that(s)?
Anhedonia (loss of interest)	Markedly diminished interest or pleasure in all, or almost all, previously enjoyable activities.	Believe that your/your colleagues' job stressors made you or your colleagues feel like that?
Sleep alteration	Insomnia (difficulty falling asleep or staying asleep) or hypersomnia (excessive sleep) nearly every day.	Believe that your or your colleagues' job stressors made it difficult to sleep?
Fatigue/loss of energy	Persistent feelings of fatigue or loss of energy, even after minimal exertion.	Believe that you/ your colleagues felt exhausted because of job stressors?
Appetite alteration	Significant changes in appetite, either a decrease or an increase.	Believe that your/your colleagues' job stressor brought that?
Feelings of worthlessness	Feelings of worthlessness or excessive or inappropriate guilt that may be delusional.	Believe because your experience or your colleagues' experience at work made you/him feel like a failure?
Cognitive impairment	Reduced ability to think or concentrate, or indecisiveness, nearly every day.	Had your or your friend's job stressed you/him so much that you/ your friend (health worker) had trouble focusing on what you/your colleagues were doing?
Psychomotor Agitation or Retardation	Observable psychomotor agitation or retardation, such as restlessness or slowed movements, that is noticeable by others	Because of your/ your colleagues' job stressor that, noticeably slowed you down for example doing your job or talking to your friend?
Suicidal ideation	Recurrent thoughts of death, suicidal ideation without a specific plan, a suicide attempt, or a specific plan for committing suicide.	Did you or your colleagues think or may think that you or your colleagues would rather be dead than continue in this job?
**NB:** Probing continued for those health workers who could provide at least one symptom, otherwise stopped and proceeded to the subjective link between work stressors including WASH-related stressors and depression.		
